# Screen-Printed Piezoelectric Sensors on Tattoo Paper Combined with All-Printed High-Performance Organic Electrochemical Transistors for Electrophysiological Signal Monitoring

**DOI:** 10.1021/acsami.3c10299

**Published:** 2023-11-29

**Authors:** Anatolii Makhinia, Valerio Beni, Peter Andersson Ersman

**Affiliations:** † RISE Research Institutes of Sweden, Digital Systems−Smart Hardware−Printed, Bio- and Organic Electronics, 60233 Norrköping, Sweden; ‡ Laboratory of Organic Electronics, Department of Science and Technology, Linköping University, 60221 Norrköping, Sweden

**Keywords:** piezoelectric sensor, OECT, aerosol jet printing, screen printing, PEDOT:PSS, printed electronics

## Abstract

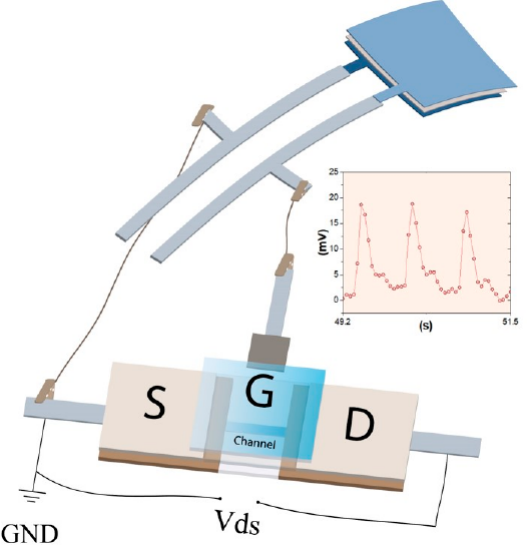

This work demonstrates sensitive and low-cost piezoelectric sensors on skin-friendly, ultrathin, and conformable substrates combined with organic electrochemical transistors (OECTs) for the detection and amplification of alternating low-voltage input signals. The fully screen-printed (SP) piezoelectric sensors were manufactured on commercially available tattoo paper substrates, while the all-printed OECTs, relying on an extended gate electrode architecture, were manufactured either by solely using SP or by combining SP and aerosol jet printing (AJP) on PET substrates. Applying a low-voltage signal (±25 mV) to the gate electrode of the SP+AJP OECT results in approximately five times higher current modulation as compared to the fully SP reference OECT. The tattoo paper-based substrate enables transfer of the SP piezoelectric sensor to the skin, which in turn allows for radial pulse monitoring when combined with the SP+AJP OECT; this is possible due to the ability of the conformable sensor to convert mechanical vibrations into voltage signals along with the highly sensitive current modulation ability of the transistor device to further amplify the output signal. The results reported herein pave the way toward all-printed fully conformable wearable devices with high sensitivity to be further utilized for the real-time monitoring of electrophysiological signals.

## Introduction

In recent decades, the field of printed electronics (PE) has sparked an interest among researchers due to scalability of manufacturing and materials, multiple choices of low-cost manufacturing methods (analog and digital), and the possibility of integration on various flexible substrates.^1^

Screen printing (SP) technology has attracted great interest due to its ease of manufacturing and high scalability; therefore, this technique has been applied in the fabrication of various organic electronic devices for a broad range of applications, e.g., biosensors, logic circuits, and amplifiers based on organic electrochemical transistors (OECTs), piezoelectric sensors, electrochromic displays, etc.^2−5^ In particular, screen printing has been utilized to create sensors for electrophysiological signal monitoring. A few years ago, Yang et al. and Zhao et al. used manual screen printing to demonstrate interdigitated or similar two-electrode structures on flexible paper-based substrates and nylon fabric, respectively.^6,7^

Piezoelectric sensors manufactured by analog and digital printing technologies on flexible substrates, such as textiles, have been reported previously for various applications, e.g., healthcare monitoring and energy harvesting.^8−13^ The demand for wearable and affordable sensors for heartbeat monitoring is important for the detection of early signs of cardiovascular diseases.^14^ Monitoring of vital signs, such as heartbeat, might reveal early signs of arrhythmia, which encompasses a large variety of disorders and abnormalities related to the heartbeat and rhythm.^15^ Ultrathin and wearable sensors that are realized solely with scalable additive printing technology are good candidates for cost-efficient monitoring of such vital signs.

In 2021, monitoring of the arterial pulse rate was demonstrated using a self-powered piezoelectric sensor based on an interdigitated structure on Parylene-C; the device was fabricated via a mix of deposition techniques, i.e., chemical vapor deposition (CVD), inkjet printing, and automatic wire bar coating.^16^ In 2022, the same group reported on arterial pulse wave monitoring with a highly sensitive device manufactured by using a similar substrate and fabrication strategy.^17^ Despite the sensitivity in the resulting devices, the proposed manufacturing process, based on the combination of CVD and printing techniques, has some drawbacks when it comes to scalability, manufacturing cost, and device footprint.

In the past decade, various applications utilizing the OECT technology have been facilitated by simplified device architectures, often allowing for fabrication via additive printing techniques.^18^ The selection of fabrication (printing) technology determines the OECT characteristics with respect to, e.g., design flexibility, manufacturing yield, transistor footprint, and printing resolution.^19,20^ The latter governs the channel dimensions (width (*W*), length (*L*), and thickness (*t*)), where downscaling implies improved OECT switching performance and increased sensitivity to the applied gate voltage. Aerosol jet printing technology (AJP) is a digital printing technique that allows high-resolution printed features of 10–20 μm on planar and curved substrates.^21^ The use of AJP alone or in combination with, e.g., photolithography or stencil printing has been reported for the fabrication of electrolyte-gated transistors with superior performance.^22,23^

The OECT is a device with three terminals, where the channel and the gate electrode are ionically bridged through an electrolyte, while the source and drain electrodes are electronically connected by an organic semiconductor material serving as the channel, e.g., the conducting polymer poly(3,4-ethylenedioxythiophene) doped with poly(styrenesulfonate) (PEDOT:PSS). This material is commercially available in various ink formulations and exhibits high ionic and electronic conductivity; hence, high current throughput and relatively short switching times are ensured in OECT devices relying on bulk charge transport.

Herein, the combination of screen and aerosol jet printing (SP+AJP) has been explored for the manufacturing of OECTs. As a result, the OECT channel area (*W* × *L* is approximately 20 × 90 μm) of the SP+AJP OECTs is considerably smaller (7.5×) as compared to fully screen-printed (SP) OECTs (*W* × *L* is approximately 150 × 90 μm), and the lowered device capacitance results in lower charge consumption upon switching the OECTs (see Figure S1 in the Supporting Information).

**Figure 1:**
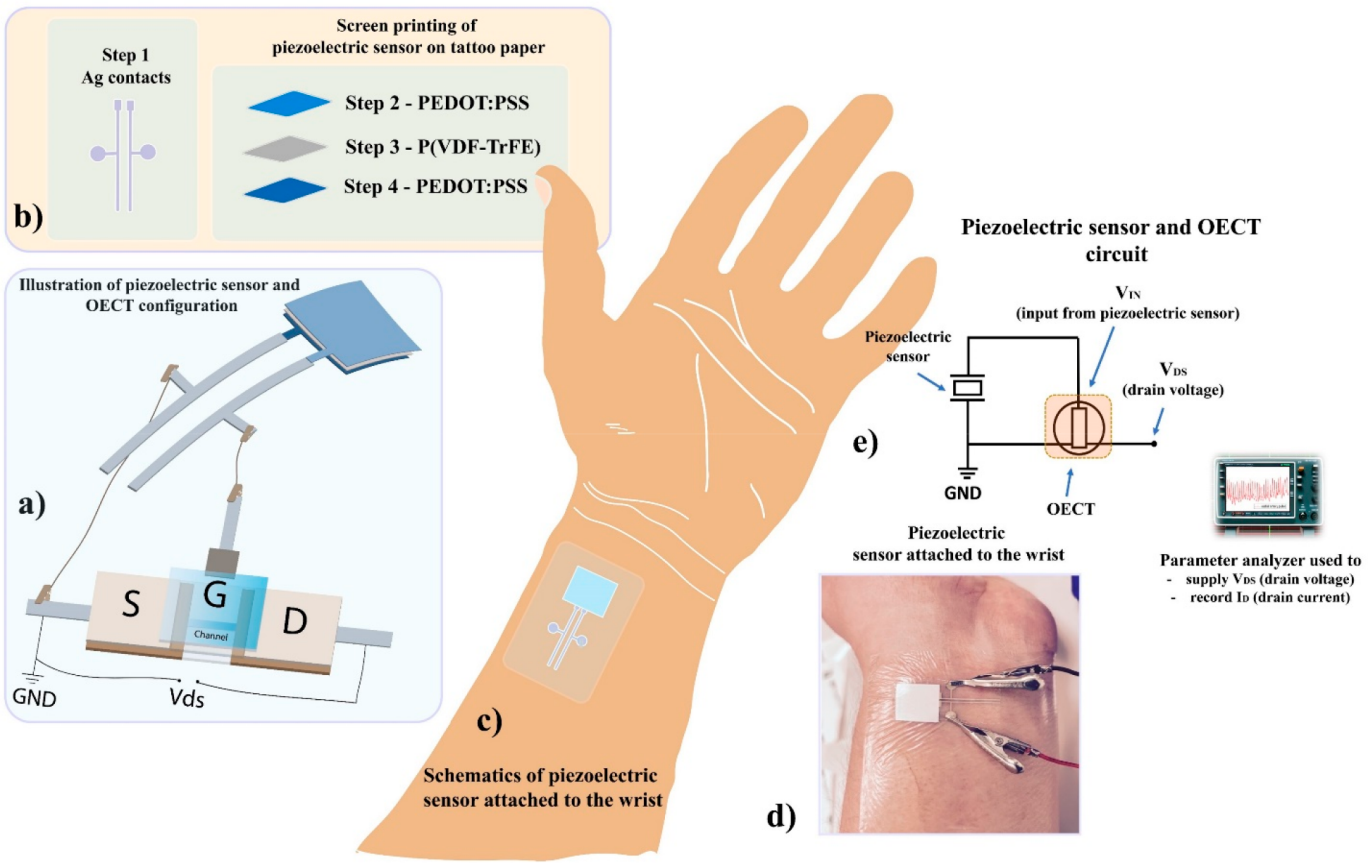
(a) Schematic layout of the all-printed sensor device obtained when combining the piezoelectric sensor (top) and the OECT (bottom). (b) Illustration of the manufacturing process of piezoelectric sensors screen-printed on tattoo paper substrates. (c) Schematic and (d) photograph demonstrating the piezoelectric sensor screen-printed on a tattoo paper substrate. (e) Schematic of the circuit used for radial pulse monitoring.

In the work reported herein, we demonstrate highly conformable and fully SP piezoelectric sensors realized on thin commercially available tattoo paper (TP) substrates. This approach utilizes a vertically stacked arrangement of the electrodes and the active layer (sandwich structure) to minimize the footprint of the devices. Process scalability is ensured by solely relying on SP, while the use of TP-based substrates allows for, as compared to PET-based substrates, ultrathin and conformable sensors. In addition, the conformability of the TP-based substrate improves the signal-to-noise ratio. Combining the SP+AJP OECT (manufactured on PET) and the TP-based piezoelectric sensor enables monitoring of low-amplitude voltage signals generated by the sensor upon exposure to the mechanical vibrations of the radial pulse, see Figure 1. In the SP piezoelectric sensors reported herein, the conventional PET substrate is replaced with the TP transfer film as the carrier, not only to enhance the conformability of the targeted on-skin wearable application (Figure 1d) but also to obtain a higher signal-to-noise ratio upon converting mechanical actions into voltage signals, see Figures S2 and S3 in the Supporting Information.

To demonstrate and evaluate the performance of the proposed concept, the radial pulse was monitored by attaching the SP piezoelectric sensor to the wrist, followed by electrically connecting it with the gate electrode of the SP+AJP OECT device.

In the long term, monolithic device integration could turn such all-printed devices into the core sensing element of a sensor patch targeting continuous monitoring of the heartbeat, which would aid timely reactions toward abnormality signs associated with the heartbeat rate, e.g., to adjust the dose of medicaments.

## Results and Discussion

### Fabrication and Characterization of Piezoelectric Sensors Screen-Printed on Tattoo Paper Substrates

A sustainable, affordable, and ultrathin (several micrometers) TP transfer film was chosen as the substrate for the fabrication of the piezoelectric sensors.

The powerful yet simple, reliable, and scalable SP technique was adopted for manufacturing the piezoelectric sensors, which comprises four printing steps. As shown in Figure 1a, the typical piezoelectric sensor material stack consists of Ag-based conductors and contact pads as well as PEDOT:PSS-based bottom and top electrodes sandwiching a piezoelectric layer based on poly(vinylidene fluoride-*co*-trifluoroethylene) (P(VDF-TrFE)).

The ferroelectric performance of the manufactured TP-based piezoelectric sensors was acquired via hysteresis loop measurements by gradually sweeping the voltage in the range ±500 V (Figure S4); the estimated remanent polarization (Pr) and the coercive electric field (Ec) are summarized in Table 1. The measured Pr and Ec values were comparable to those reported in the literature.^5,17^

**Table 1:** Ferroelectric Performance of P(VDF-TrFE)-Based Piezoelectric Sensors

reference	substrate	fabrication technology	average remanent polarization [μC cm^–2^]	average coercive field [V μm^–1^]
this work	tattoo paper (transfer film)	screen printing	7.8	50.6
(17)	Parylene-C	CVD, inkjet printing, bar coating	7.5 ± 0.2	46.9 ± 8.1
(5)	PET	screen printing	7.1	50

Fully SP TP-based piezoelectric sensors, each one with an active area of 64 mm^2^ (Figure 1), were further tested by using an actuator setup, developed in house, including also a commercial piezoelectric sensor, to quantitatively estimate the voltage response upon stable, repetitive mechanical impact.^24^ The mechanical impact was repeatedly obtained by the linear actuator at a frequency of 3 Hz (similar to the radial pulse rate), where the skin contact was mimicked by attaching a piece of natural leather. To evaluate the SP TP-based piezoelectric sensor, its voltage response was compared with the signal obtained from both a commercial piezoelectric sensor and a SP piezoelectric sensor on PET (the latter is shown in Figure S5); the three different types of sensors were subjected to tapping from the linear actuator.

**Figure 2:**
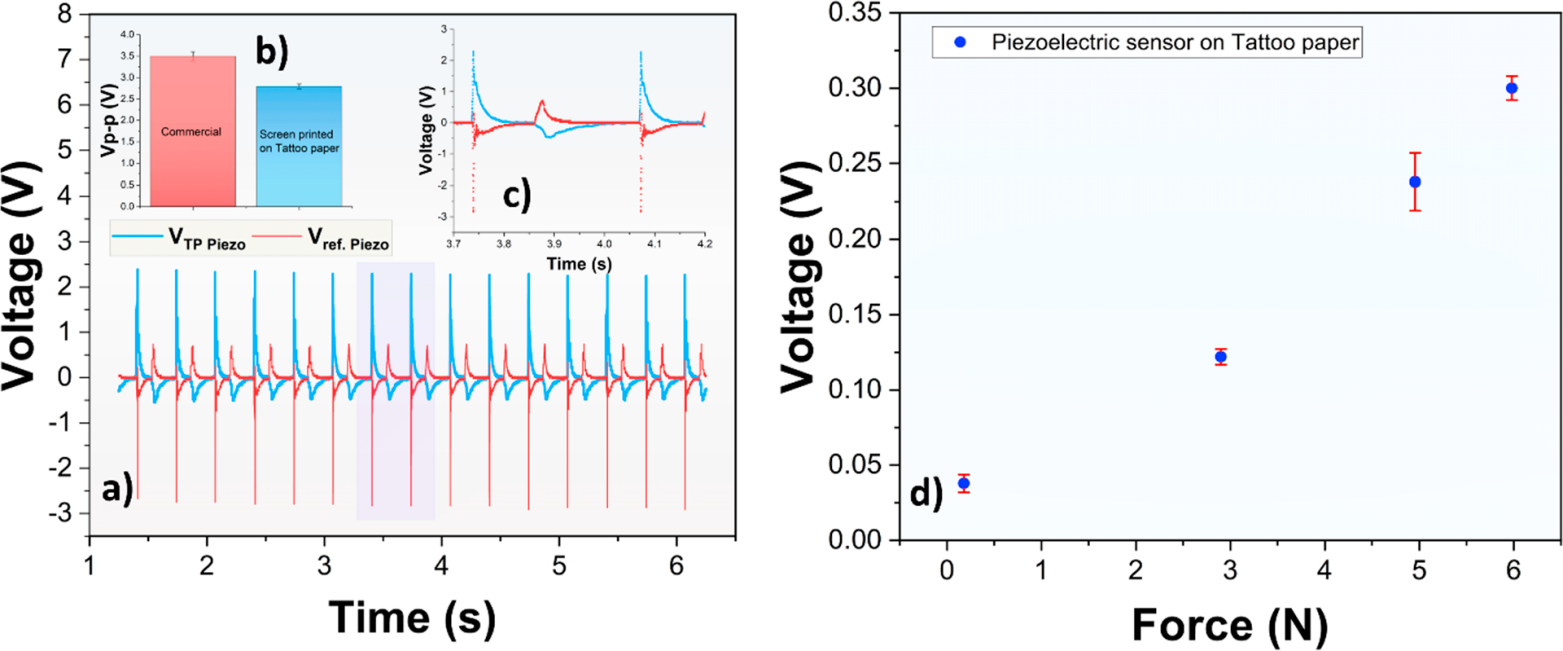
(a) Comparison of voltage output signals generated by a commercial (red) and a SP tattoo paper-based (blue) piezoelectric sensor upon repetitive tapping. (b) The distributions of the peak-to-peak voltage output signals generated by the piezoelectric sensors upon repetitive tapping are presented. (c) Zoom-in of the same data already shown in a. (d) Voltage output signals as a function of the force applied to the SP TP-based piezoelectric sensor.

Figure 2a shows the output voltage signals as a function of time, with average peak-to-peak voltage amplitudes of 3.5 ± 0.1 and 2.8 ± 0.06 V (inset Figure 2b), generated by the commercial and SP TP-based piezoelectric sensors upon repetitive tapping with the same force.

The comparison between the SP TP-based and commercial piezoelectric sensors reveals that the output voltage response is repetitive and stable over time (Figure 2a and 2c). Figure 2d shows the voltage output signals as a function of force, measured by the linear actuator setup. The force and the voltage were concurrently acquired upon tapping the SP TP-based piezoelectric sensor; at least three measurements were recorded for each data point. The TP-based piezoelectric sensors were capable of detecting forces as low as 0.18 N, which is comparable with the piezoelectric sensors reported on Parylene-C.^17^ The voltage amplitudes generated upon applying such low force correspond to approximately 35–40 mV.

### Evaluation of Screen- and Aerosol Jet-Printed OECTs for Radial Pulse Monitoring

The novel fabrication approach based on the combination of SP and AJP for the manufacturing of OECTs and OECT-based logic circuits has been previously demonstrated and reported.^19^ The selection of an appropriate deposition technique is, besides the choice of materials, a key for tuning the device dimensions to optimize and improve the performance, e.g., switching time, transconductance, ON/OFF ratio, and design flexibility for high-throughput manufacturing.^20^

The use of AJP allowed one to considerably reduce, when compared to fully SP devices, the width of the channel (from 150 to 20 μm) and hence the overall area of the channel. This resulted in a lower device capacitance, which led to a reduced charge consumption upon switching (see Figure S1 in the Supporting Information), high transconductance (3.9 mS or 190 S m^–1^), and shorter switching times (∼1 ms) in the OECTs.^19^

**Figure 3:**
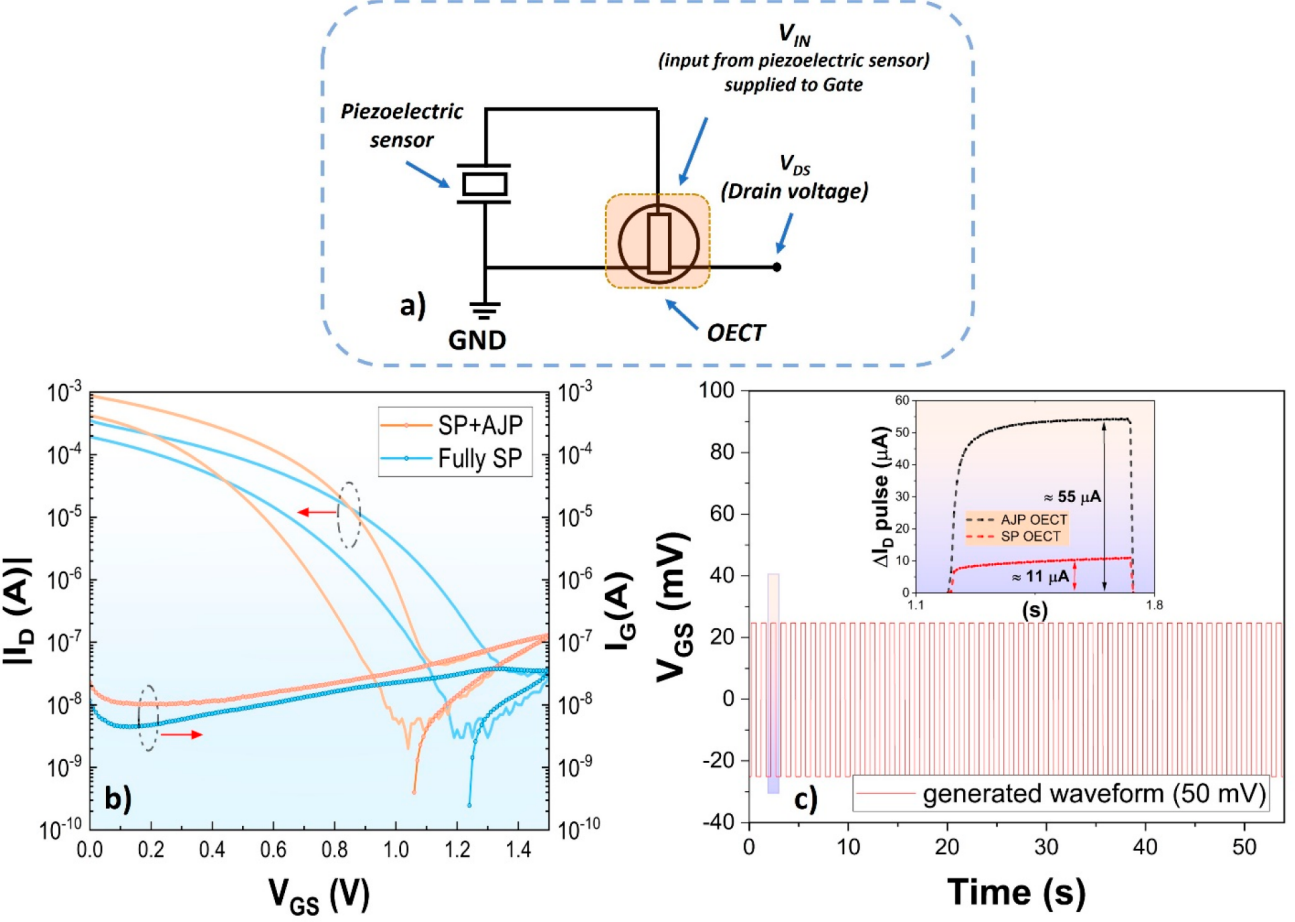
(a) Schematic of the circuit used to record the current modulation when connecting an OECT with either a piezoelectric sensor attached to the wrist or a function generator. Comparison of (b) transfer and (c) dynamic switching characteristics of all-printed OECTs having either SP or AJP channels. The inset of Figure 3c shows the zoom-in of the dynamic data between approximately 1.2 and 1.7 s. *V*_DS_ = −1 V in both transfer and dynamic measurements.

**Figure 4:**
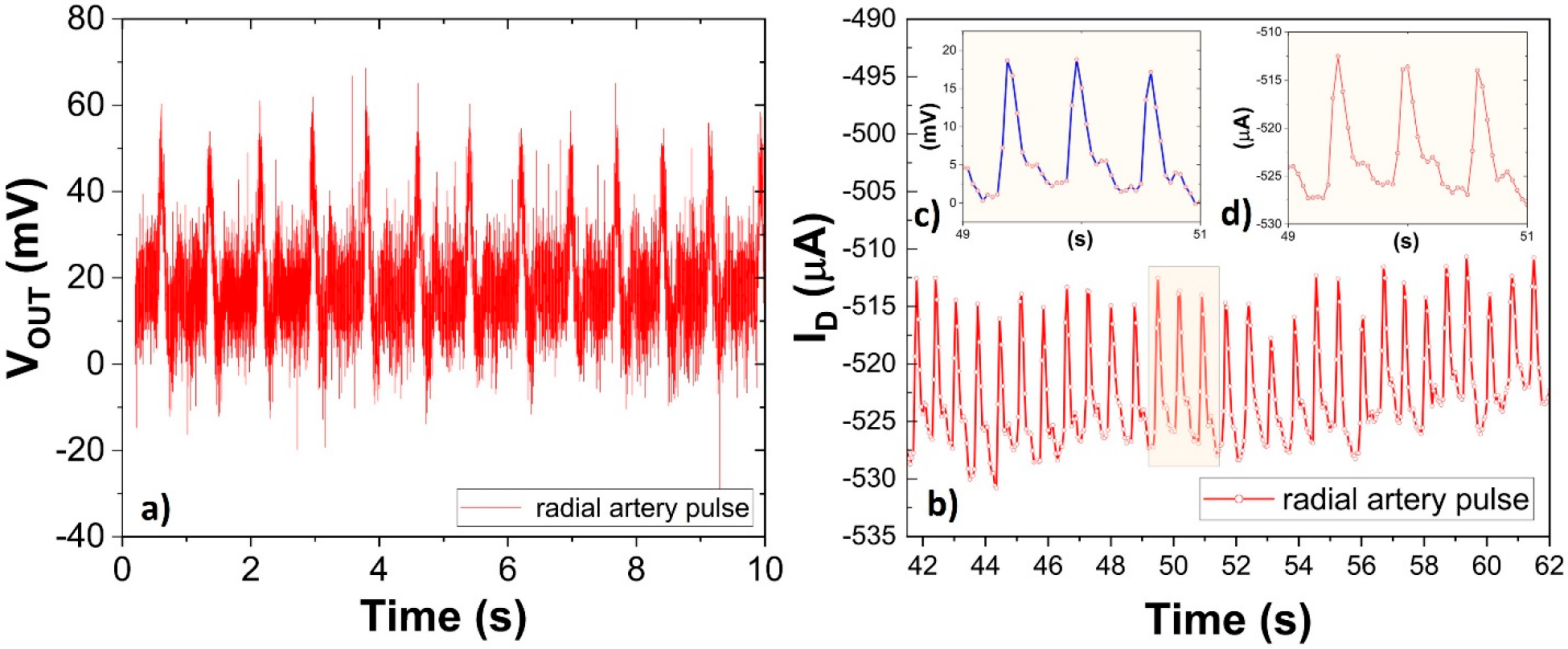
(a) The output voltage signal generated by the TP-based SP piezoelectric sensor when exposed to the radial artery pulse. (b) Alternating drain current recorded in the SP+AJP OECT as a function of time when connecting the OECT gate electrode with the output voltage signal of the TP-based SP piezoelectric sensor. (Insets c and d) Gate voltage generated by the TP-based SP piezoelectric sensor and the resulting drain current modulation of the OECT, respectively, as a function of the pulsing artery wave during the time interval from 49 to 51 s.

Figure 3a shows a simple circuit that connects a piezoelectric sensor with an OECT to monitor the current modulation induced by the mechanical vibrations of the radial pulse on the wrist. Note that the mechanical vibrations of the radial pulse generate voltage signal amplitudes of at least 35–40 mV from the piezoelectric sensor (Figure 4a), which is at least equal to the signals obtained upon tapping at the force detection limit (0.18 N). The transfer characteristics of fully SP and SP+AJP OECT devices are shown in Figure 3b. These devices are differing with respect to several parameters: the ON current (*I*_ON_), ON/OFF ratio, and gate voltage required to fully switch off the channel to its reduced state (*V*_G,OFF_), i.e., *V*_G,OFF_ −1.16 V and an ON/OFF ratio of ∼2.1 × 10^4^ are obtained for SP+AJP OECTs, while *V*_G,OFF_ −1.37 V and an ON/OFF ratio of ∼1 × 10^4^ are obtained for the fully SP OECTs. Figure 3c instead shows the dynamic measurement of an OECT with a 50 mV (peak-to-peak, zero voltage offset) square wave signal applied to the gate electrode. The 50 mV waveform, acquired from the built-in function generator of an oscilloscope, results in ±25 mV pulses applied to the gate electrodes of the OECT devices; this was intended to mimic the low-voltage amplitude signals provided by the TP-based piezoelectric sensor (Figure 4a) when subjected to the mechanical stimulation of the radial artery pulse.

The inset of Figure 3c shows the drain current modulation upon applying ±25 mV at the gate electrodes of the fully SP and SP+AJP OECT devices, where the latter shows a factor of 5 higher current modulation. This is explained by the relatively smaller area of the AJP channel, resulting in lower device capacitance, lower charge consumption, and, hence, increased device sensitivity, see Figure S1. This is an important result highlighting the potential of utilizing SP+AJP manufactured OECT devices for the monitoring of voltage signals with low amplitudes in addition to the shortened switching times demonstrated previously.

Figure 4a shows the low amplitudes of the alternating output voltage (*V*_OUT_) signal (Figure S2 shows the same measurements with and without a low-pass filter) generated by the TP-based piezoelectric sensor as a function of time when attached to the wrist. The alternating *V*_OUT_ signal corresponds to the radial artery pulse rate. As demonstrated by the dynamic measurement in Figure 3c, the SP+AJP OECT device outperformed the fully SP OECT in terms of current modulation. Therefore, to evaluate the possibility of using all-printed OECTs for recording of the output voltage signal of the TP-based piezoelectric sensor, the latter was connected to the gate electrode of the SP+AJP OECT device. As shown in Figure 4a, the TP-based piezoelectric sensor generates 40–60 mV voltage amplitude peaks (unfiltered, high-frequency noise), while when connected with an OECT device, it results in clearly distinguishable peaks (improved signal-to-noise ratio) but lower in amplitude (similar to when applying the low-pass filter in Figure S2). The alternating *V*_OUT_ of 15–18 mV (Figure 4c) resulted in a drain current modulation of 12–15 μA (Figure 4d). The lower amplitudes of the *V*_OUT_ pulse in Figure 4c can be explained by the fact that the OECT acts as a low-impedance device when compared with the high-impedance measurement carried out by the oscilloscope in Figure 4a.

The monitoring of the *V*_IN_ (from piezoelectric sensor) signal by using the SP+AJP OECT device (Figure 4b) resulted in a significant reduction of the high-frequency noise contribution (Figure 4c) as compared to the *V*_OUT_ of the standalone piezoelectric sensor (Figure 4a). This result is explained by the lower sampling rate upon recording the drain current (50 ms), while a sampling rate of 160 μs was used in the oscilloscope measurements.

Additionally, the circuit shown in Figure S6 was used to convert the current measurement mode into monitoring of an output voltage signal, aiming at a comparison of the difference in voltage amplification between SP+AJP and fully SP OECTs. The results show that the voltage amplification of the SP+AJP OECT is approximately 2.5 times higher, as compared to the fully SP OECT, upon monitoring the radial pulse by the TP-based piezoelectric sensor, see Figure S7. It should be noted that the OECTs used in this voltage amplification test were manufactured about 1 year ago, and the polarization of the TP-based piezoelectric sensor was performed approximately 6 months ago, both indicating the robustness of these devices upon storage in ambient conditions.

## Conclusion

In this work, for the first time, we demonstrate piezoelectric sensors manufactured by screen printing (SP) on commercially available tattoo paper (TP) substrates for seamless integration in healthcare applications. Combining the SP piezoelectric sensor on TP with an all-printed OECT, in which the channel is deposited via aerosol jet printing (AJP), results in a sensor device capable of monitoring low-voltage amplitudes. The ability of fully SP and SP+AJP OECTs to record low-amplitude square wave voltage signals was first assessed; the OECT devices with AJP channels showed five times higher current modulation as compared to OECTs with SP channels. Similarly, the voltage amplification differed by a factor of 2.5, in favor of the SP+AJP OECT. This significant improvement in performance is explained by the lowered device capacitance of the AJP OECT channels. To further verify the concept, a TP-based piezoelectric sensor and an OECT with an AJP channel were configured into an all-printed sensor device to allow for artery radial pulse monitoring. The results demonstrate the potential of incorporating SP piezoelectric sensors with all-printed OECTs for monitoring of low-voltage amplitude signals, e.g., the radial pulse. Hence, this study provides a fast, low-cost, and scalable method for the fabrication of all-printed, ultrathin, and conformable sensor solutions for enhanced user experiences.

## Experimental Section

### Screen Printing of Piezoelectric Sensors on Tattoo Paper Substrate

The manufacturing of the piezoelectric sensors included several consecutive screen printing steps performed in ambient controlled conditions (∼22 °C and ∼50% RH). The commercially available tattoo paper (Gecko tattoo transfer paper) was used as the substrate. DEK Horizon 03iX screen printing equipment was used for the deposition of all layers. The fabrication of the respective piezoelectric sensor comprised 6 sequential layers. Step 1 (optional): insulating layer (UVSF, purchased from Marabu) to increase the manufacturing yield of the printed sensors. Step 2: Ag-based conductors and contact pads (Ag 5000 paste, purchased from DuPont). Steps 3 and 5: PEDOT:PSS-based (Clevios S V4 paste, purchased from Heraeus) bottom and top electrodes. Step 4: P(VDF-TrFE) (FC 20 INK P, purchased from Piezotech). Step 6 (optional): insulating layer for mechanical protection (UVSF, purchased from Marabu).

The printed layers in steps 2, 3, 4, and 5 were thermally treated (100–120 °C) in either a convection or a conveyor belt oven, while the printed layers in steps 1 and 6 were cured by exposure to ultraviolet light.

### Screen and Aerosol Jet Printing of OECTs

The fabrication of all-printed OECTs was carried out in ambient controlled conditions (∼22 °C and ∼50% RH). Polyethylene terephthalate (PET) (Polifoil, purchased from Policrom) was chosen as the plastic substrate. All of the screen-printed layers were deposited using DEK Horizon 03iX screen printing equipment. The aerosol jet-printed OECT channels were deposited by using an aerosol jet printer (Optomec AJ 200 system) embedded in a Ceraprinter (F-serie, purchased from Ceradrop). The printing steps and related information on the OECT manufacturing process have been previously reported.^19^

### Force Measurement Setup

The measurement setup included a linear actuator with natural leather attached, a scale, and an oscilloscope. The mechanical impact (tapping) was obtained by the linear actuator from Miyachi Unitek. Upon tapping, the weight (grams converted into Newton) and the voltage were acquired via the scale (Soehnle) and the oscilloscope (DSOX1204G from Keysight).

### Tapping Measurement Setup

The automated setup included a linear actuator with natural leather attached to mimic the skin contact. The adjustable stage of the setup, with the piezoelectric sensor attached to its plate, allows one to set the distance between the piezoelectric sensor and the linear actuator with a micrometer screw. The plate with the attached TP-based SP piezoelectric sensor was also equipped with a commercial piezoelectric sensor (purchased from DT Sensors) to provide a quantitative evaluation of the relative impact force.^24^ The frequency of the linear actuator was controlled by adjusting the voltage waveform generated by the built-in function generator of the oscilloscope.

### Electrical Characterization

All of the measurements related to piezoelectric sensors and OECTs were carried out in ambient controlled conditions (∼22 °C and ∼50% RH). The characterization of the ferroelectric properties (polarization curve) was performed by sweeping the voltage in the range ±500 V. A digital storage oscilloscope (DSOX1204G from Keysight) was used together with the force measurement and tapping setups to record the output voltage signals generated by the piezoelectric sensors. To filter the *V*_OUT_ signals, either a built-in (oscilloscope) or software-based (Origin) low-pass filter (5 Hz) was used. The transfer (*I*_D_ vs *V*_GS_) and dynamic (*I*_D_ vs time) characteristics of the OECTs were performed using a semiconductor parameter analyzer (HP/Agilent 4155B). For recording the radial pulse (Figure 4), a sampling rate of 50 ms (resulting in a reduction of high-frequency noise) and 160 μs was employed for the semiconductor parameter analyzer and the oscilloscope, respectively. In transfer and dynamic measurements, the drain voltage was set to −1 V. The gate voltage (50 mV peak-to-peak, zero offset voltage) for the dynamic measurements was generated by the built-in function generator of the oscilloscope, while *V*_G_ was generated by the piezoelectric sensor and recorded by the semiconductor parameter analyzer in the demonstration of the radial pulse monitoring. The human wrist skin was covered with a transparent adhesive film (Tegaderm from 3M) prior to attaching the piezoelectric sensor and following recording of the radial pulse. Additionally, the subject provided a written consent for the radial pulse measurements.

## Data Availability

The data underlying this study are openly available in Zenodo at https://doi.org/10.5281/zenodo.10213915.

## Supporting Information Available

OECT charge consumption; voltage output signal from a tattoo paper-based piezoelectric sensor upon radial pulse measurements; voltage output signal from a PET-based piezoelectric sensor upon radial pulse measurements; typical polarization–electric hysteresis curve obtained from piezoelectric sensors screen-printed on tattoo paper substrates; voltage output signal from a PET-based piezoelectric sensor upon repetitive tapping; circuit schematic for the OECT voltage measurement mode; difference in voltage amplification when comparing different OECTs (PDF)
