# Supplementary material for: Screen-Printed Piezoelectric Sensors on Tattoo Paper Combined with All-Printed High-Performance Organic Electrochemical Transistors for Electrophysiological Signal Monitoring
Source: ACS Appl Mater Interfaces. 2023 Nov 29;16(45):61428–34. doi: 10.1021/acsami.3c10299 (PMC11565470; doi:10.1021/acsami.3c10299)
Supplement: Supplementary file 1 — am3c10299_si_001.pdf [file am3c10299_si_001.pdf]

Supporting Information

Screen-Printed Piezoelectric Sensors on Tattoo Paper Combined with All-Printed High Performance Organic Electrochemical Transistors for Electrophysiological Signal Monitoring

Anatolii Makhinia,[†,‡] *Valerio Beni*[†] *and Peter Andersson Ersman*[*,†]

[†] RISE Research Institutes of Sweden, Digital Systems–Smart Hardware–Printed, Bio- and Organic Electronics, 60233 Norrköping, Sweden

[‡] Laboratory of Organic Electronics, Department of Science and Technology, Linköping University, 60221 Norrköping, Sweden

Email: peter.andersson.ersman@ri.se.

Measurement of the charge required in OECT transient measurements

The charges required for OECT transient measurements (when V_G switches from 1.5 to 0 V, to minimize parasitic drain current contributions from the carbon-based source and drain electrodes) significantly differ for the two different OECT manufacturing approaches. Approximately 47 nC was required to switch the fully SP OECT device from OFF to ON (Figure S1a), while only ~12 nC was required to switch the SP+AJP OECT (Figure S1b).

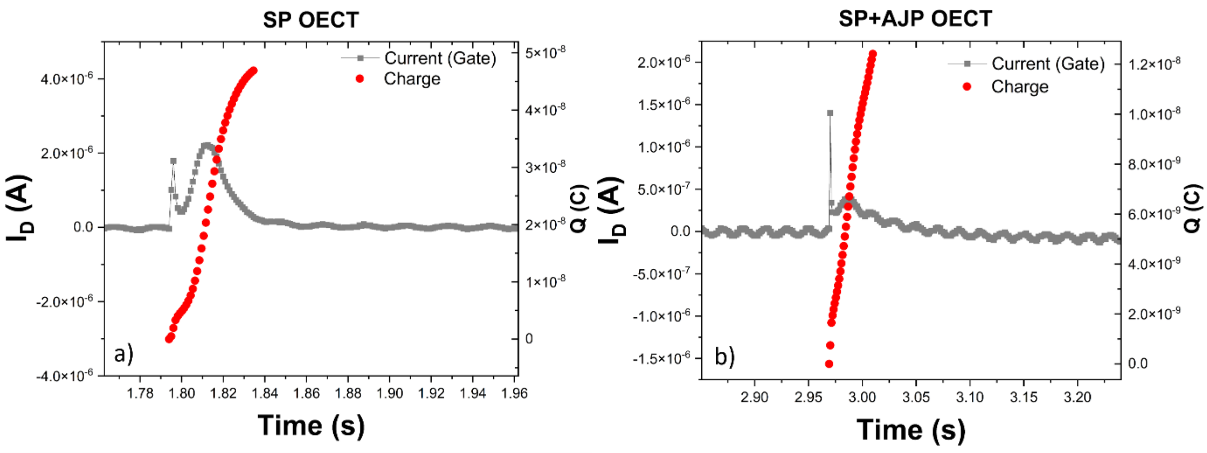

Figure S1. Current versus time measurements (V_D = -1 V, V_G switched from 1.5 to 0 V) to define the amount of charge required to switch the a) fully SP and the b) SP+AJP OECT devices to their ON states. The time between two consecutive data points is ~2 ms in both measurements.

Measuring radial artery pulse with tattoo paper- and PET-based screen-printed piezoelectric sensors

Triggering the tattoo paper-based screen-printed piezoelectric sensor by the mechanical impact from the radial artery pulse (Figure S2) resulted in 40-60 mV output voltage signals with high frequency noise, while the signal amplitudes were lowered to 20-25 mV after applying a 5 Hz low pass filter.

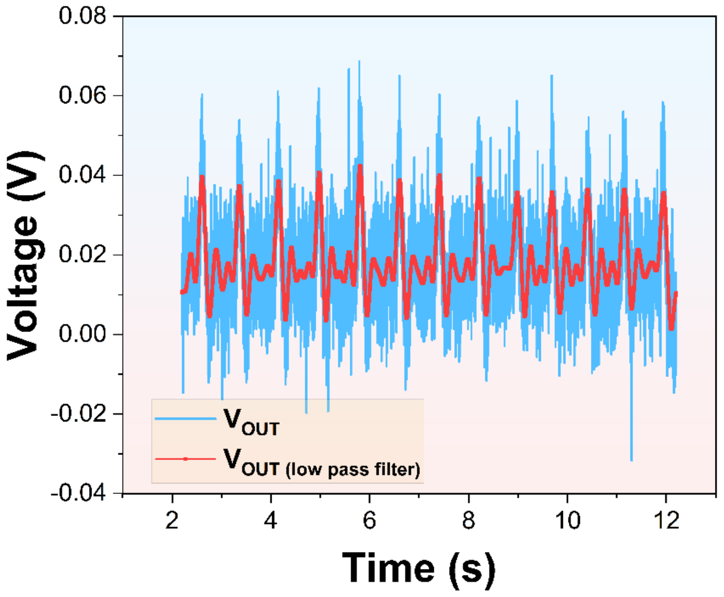

Figure S2. Comparison of filtered (5 Hz low pass filter; red graph) and non-filtered (blue graph) output voltage signals generated by the tattoo paper-based SP piezoelectric sensor upon pulsing radial artery.

The SP piezoelectric sensor on PET is also capable of monitoring the radial artery pulse, at least when pushed towards radial artery. In Figure S3, the peaks of the output voltage signal (after low pass filtering) are discernable, though with lower amplitudes and less repeatability as compared the tattoo paper-based SP piezoelectric sensor (Figure S2), which is explained by the rigidity and thickness (125 µm) of the PET substrate.

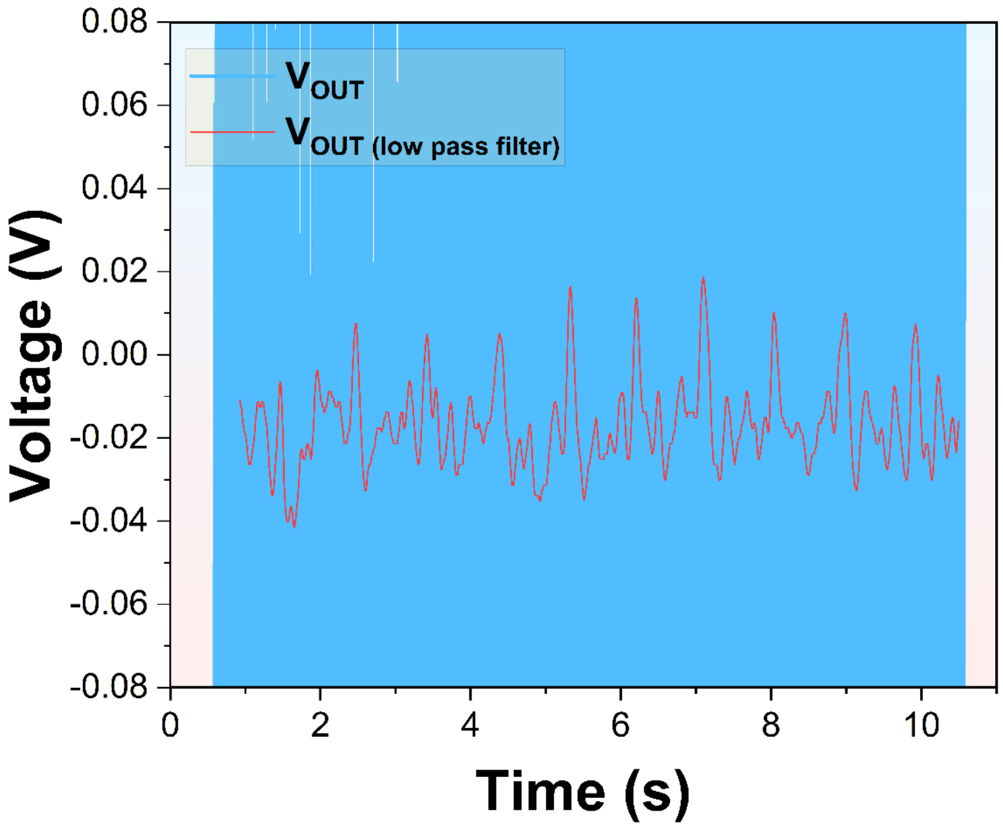

Figure S3. Comparison of filtered (5 Hz low pass filter; red graph) and non-filtered (blue graph) output voltage signals generated by the PET-based SP piezoelectric sensor upon pulsing radial artery.

Polarization-electric (P-E) hysteresis measurement

The high electric field was applied across the screen-printed piezoelectric layer starting at 0 V and in (20 V) steps increasing to +500 V, to orient the dipoles and facilitate piezoelectric activity. After that, the voltage was decreased stepwise until -500 V and then increased till 0 V again. At about 300 V and -300 V, the polarization changes direction. The voltage required to change the direction depends on the thickness of the piezoelectric layer.

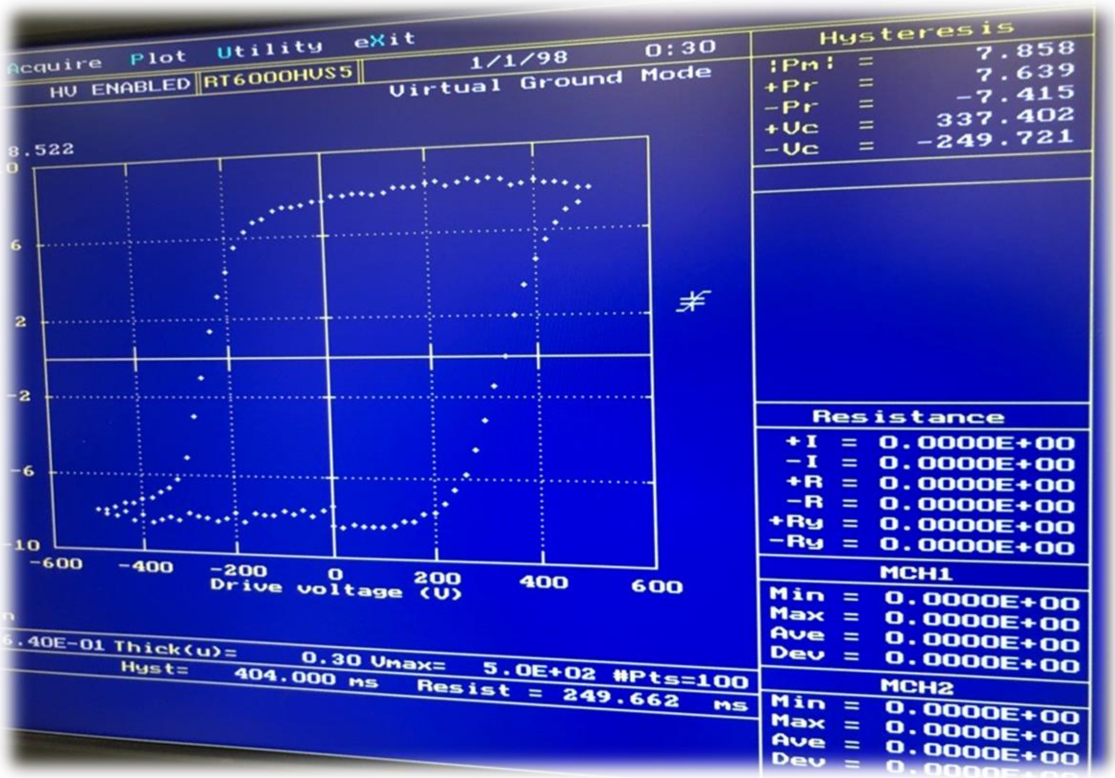

Figure S4. Photograph of the P-E hysteresis loop graph. The voltage sweeps are typically repeated a few times to optimize the polarization. Note that the polarization remained also after storing the screen-printed piezoelectric sensors in ambient conditions for approximately 6 months, i.e., the storage time did not have any impact on the device functionality.

Tapping of the PET-based screen-printed piezoelectric sensor

As shown in Figure S5, the output voltage signal of the PET-based SP piezoelectric sensor is stable over time and repetitive in shape and amplitude. Average peak-to-peak voltage amplitudes of 2.6 ± 0.02 V were recorded, and in comparison with tattoo paper-based SP piezoelectric sensors (2.8 ± 0.06 V) the PET-based device generated comparable or slightly lower voltage output amplitudes, which may be explained by the more rigid and less conformable plastic substrate.

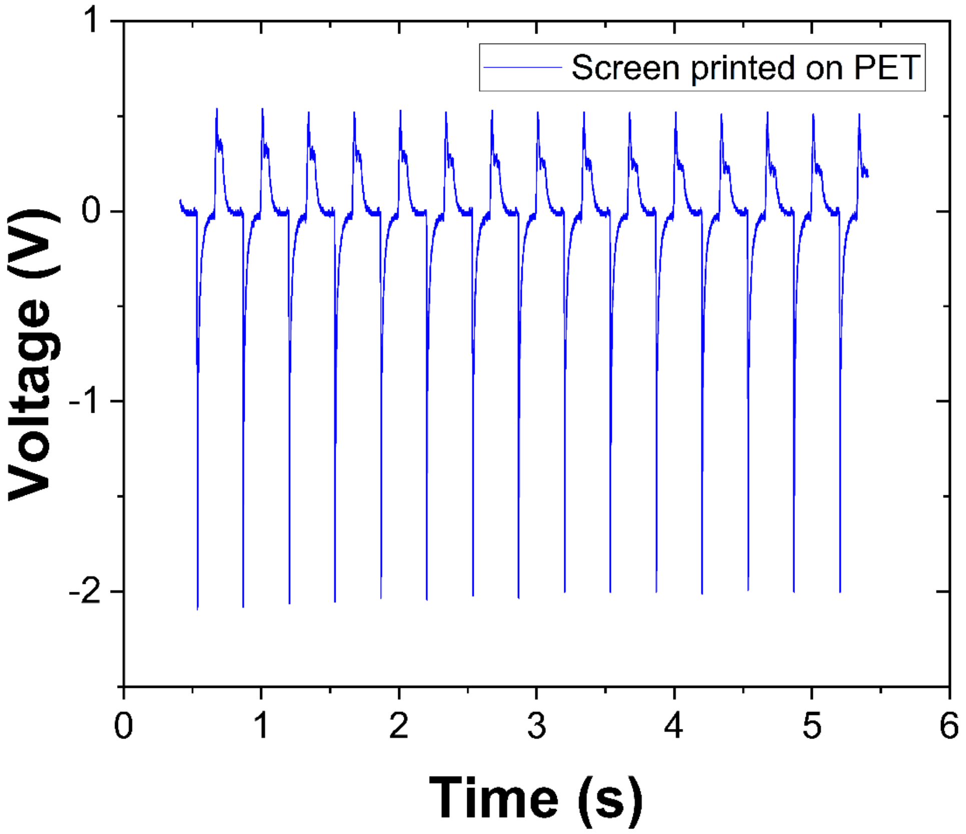

Figure S5. Voltage output signals generated by a PET-based SP piezoelectric sensor.

Circuit design for recording low voltage signals by the OECT

Figure S6 depicts a circuit schematic that enables conversion from current measurement mode (of the OECT drain current) into voltage measurement mode. This circuit is designed and tested to compare the amplification obtained in SP+AJP and fully SP OECTs. The circuit consists of a digital oscilloscope with an integrated function generator that generates an alternating 50 mV peak-to-peak voltage signal (similar to the signal obtained from the TP-based piezoelectric sensor triggered by the radial pulse), an OECT (either SP+AJP or fully SP), and a resistor (R1; 20 and 2 kΩ when using the SP+AJP and the fully SP OECT, respectively).

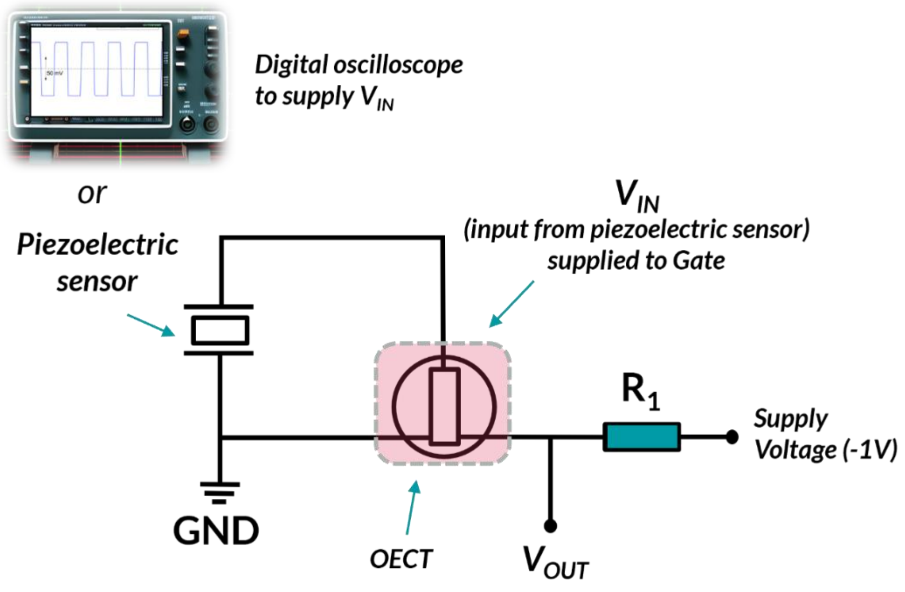

Figure S6. Circuit used to record low voltage signals applied to the gate electrode of the OECT.

Measuring voltage output signals to evaluate the OECT amplification

Figure S7 shows the voltage output levels when using either fully SP or SP+AJP OECTs connected according to the circuit exemplified in Figure S6. In relation to the input signal supplied by the function generator (50 mV), the SP+AJP OECT provides approximately 2.5 times higher amplification as compared to the fully SP OECT (~128 mV vs. ~52 mV). This difference in voltage amplification highlights the benefit of using SP+AJP OECTs in combination with piezoelectric sensors. It may be noted that the current modulation differed by a factor of 5 in the graphs shown in Figure 3. However, the lower voltage amplification factor (V_{OUT} 128 mV vs. V_{IN} 50 mV for the SP+AJP OECT) shown in Figure S7 could possibly be enhanced by optimization of the resistor value and the supply voltage used in this measurement setup.

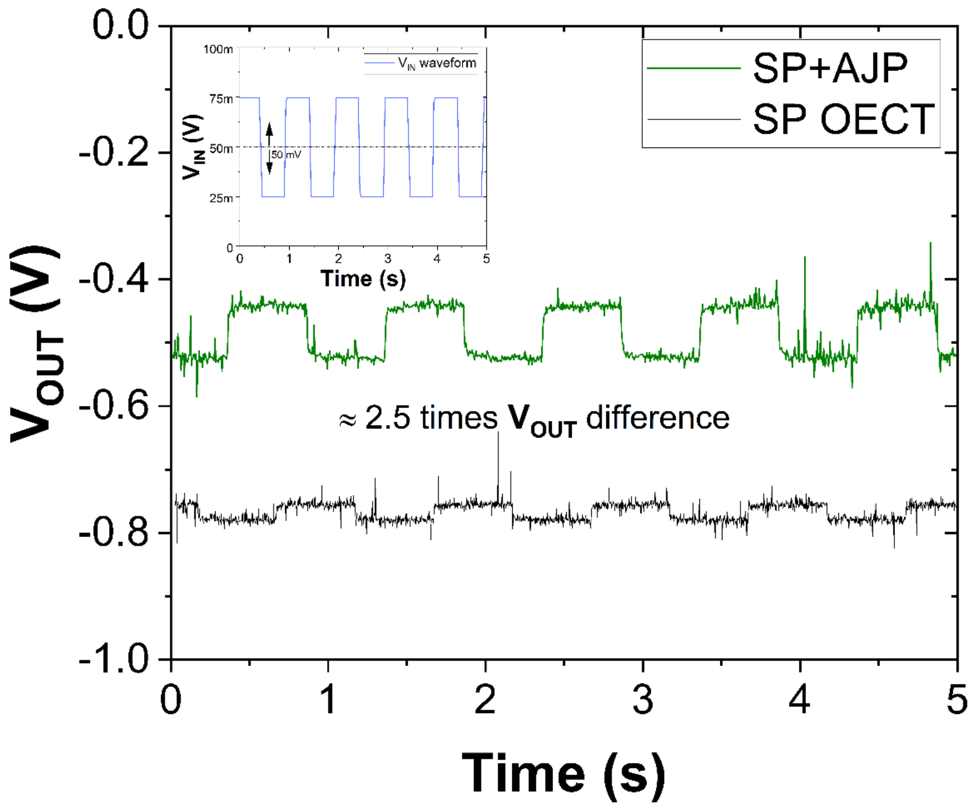

Figure S7. Voltage output signals when connecting either a fully SP (black) or a SP+AJP (green) OECT to the test circuit. The inset shows the alternating V_{IN} signal applied to the OECT gate electrodes (50 mV peak-to-peak with 50 mV positive offset).
